# Integrating PSA Change with PSA Density Enhances Diagnostic Accuracy and Helps Avoid Unnecessary Prostate Biopsies

**DOI:** 10.3390/diagnostics15162027

**Published:** 2025-08-13

**Authors:** Yi-Ju Chou, Bor-En Jong, Yao-Chou Tsai

**Affiliations:** 1 Division of Urology, Taipei Tzu Chi Hospital, Buddhist Tzu Chi Medical Foundation, New Taipei 231, Taiwan; andy0611andy@gmail.com (Y.-J.C.);; 2 School of Medicine, Tzu Chi University, Hualien 970, Taiwan

**Keywords:** PSA, PSA density, prostate cancer

## Abstract

**Background:** Prostate-specific antigen (PSA) levels can be transiently elevated in benign conditions. Therefore, guidelines recommend repeat PSA testing before a biopsy. However, PSA should be adjusted for the prostate volume to improve its predictive accuracy for prostate cancer. This study aimed to compare the diagnostic performance of the PSA density and PSA change for prostate cancer and to evaluate whether their combination can further reduce unnecessary biopsies. **Methods:** We retrospectively analyzed patients who underwent a prostate biopsy between January 2020 and December 2024. Inclusion criteria were an initial PSA level between 3 and 20 ng/mL and two PSA measurements within an eight-week interval prior to the biopsy. Patients using 5-alpha reductase inhibitors before the biopsy were excluded. Receiver operating characteristic (ROC) curves and the area under the curve (AUC) were used to compare the diagnostic performance of each predictor for prostate cancer and clinically significant prostate cancer (csPCa). **Results:** A total of 291 patients were included. Patients with prostate cancer had higher PSA levels, smaller PSA declines, and a higher PSA density. The PSA density showed a superior diagnostic accuracy compared with the PSA change for both prostate cancer and csPCa. The PSA density calculated by a transrectal ultrasound or MRI yielded a similar diagnostic performance. However, the accuracy of the PSA density decreased in patients with a large prostate volume. Incorporating a criterion of a >20% PSA decline to exclude biopsy candidates improved the performance of the PSA density and further reduced unnecessary biopsies. **Conclusions:** The PSA density demonstrates good diagnostic accuracy for predicting prostate cancer. However, incorporating the PSA change further reduces unnecessary biopsies. Therefore, combining both factors provides a more effective approach for determining the need for a prostate biopsy.

## 1. Introduction

Prostate cancer is the second most common cancer worldwide, and its prevalence increases with age [1,2]. Prostate-specific antigen (PSA) is a widely used biomarker for prostate cancer. Previous studies have demonstrated that PSA-based screening can reduce the prostate cancer-specific mortality [3]. Moreover, when diagnosed at an early stage, prostate cancer has a five-year survival rate exceeding 99%, underscoring the importance of early detection [4]. A prostate biopsy remains the standard method for confirming the diagnosis. Although the risk of complications and mortality is relatively low, a biopsy is an invasive procedure and may subject patients without cancer to unnecessary harm [5]. Therefore, it is crucial to identify patients at a higher risk of harboring cancer before proceeding to a biopsy.

While PSA is associated with prostate cancer, it may also rise in benign conditions, such as benign prostatic hyperplasia, resulting in limited specificity. Furthermore, transient PSA elevations may occur due to factors such as inflammation, ejaculation, or urinary retention [6]. Clinical guidelines recommend repeating PSA testing prior to a biopsy in cases of elevated PSA [7]. Previous studies on repeat PSA testing suggest that a decrease in PSA levels is associated with a lower risk of cancer, whereas stable PSA values are more likely to indicate the presence of malignancy [8,9,10].

However, PSA levels are closely correlated with the prostate volume [11]. In patients with larger prostates but no signs of inflammation, PSA levels may be elevated while remaining relatively stable between measurements. As a result, such PSA patterns may be misinterpreted as suggestive of a high cancer risk, potentially leading to unnecessary biopsies. This highlights the importance of incorporating the prostate volume into the decision-making process for biopsies. The PSA density, calculated by dividing PSA by the prostate volume, may reduce false-positive biopsy decisions in patients with large prostates. To date, no studies have directly compared the diagnostic performance of the PSA density with that of the PSA change in guiding biopsy decisions. Therefore, the objective of this study was to evaluate and compare the diagnostic accuracy of these two predictors. Additionally, we evaluated whether a combination of both factors could more accurately identify patients who truly require a biopsy.

## 2. Materials and Methods

### 2.1. Study Population

We retrospectively analyzed data from patients who underwent prostate biopsy between January 2020 and December 2024. Inclusion criteria were an initial PSA level between 3 and 20 ng/mL and two PSA measurements obtained within an interval of less than eight weeks prior to biopsy. Patients receiving 5-alpha reductase inhibitors were excluded. The biopsy technique was not restricted and included random, cognitive fusion, and software fusion methods. This retrospective data analysis was performed in accordance with the Declaration of Helsinki and was approved by the Institutional Review Board of Taipei Tzu Chi Hospital (No. 14-IRB044). The requirement for informed consents was waived by the Institutional Review Board of Taipei Tzu Chi Hospital due to the retrospective nature of this study.

### 2.2. Measurement of PSA and Related Parameters

All PSA values were measured using the Access^®^ Hybritech^®^ chemiluminescent immunoassay (Beckman Coulter, Brea, CA, USA). PSA change was calculated as follows: (second PSA—initial PSA)/initial PSA. The absolute PSA change was defined as the absolute value of PSA change. PSA density was calculated as initial PSA divided by prostate volume. Prostate volume was calculated with ellipsoid formula using either transrectal ultrasound (TRUS) or MRI. Prostate volume was categorized according to the EAU guidelines as small (<30 mL), medium (30–80 mL), and large (>80 mL) [12].

### 2.3. Biopsy Protocol

For patients undergoing random biopsy, a standard 12-core approach was generally adopted. The number of biopsy cores was adjusted according to the patient’s condition, such as reducing the number if the patient experienced discomfort. For cases receiving fusion biopsy, both targeted and random biopsies were performed. The number of random biopsy cores was adjusted according to the number of targeted cores obtained. The selection of targets for fusion biopsy was based on the Prostate Imaging and Reporting Data System (PI-RADS) version 2.1, and only lesions with a PI-RADS score of 3 or higher were selected for targeted biopsy.

### 2.4. Outcomes of Interest

Clinically significant prostate cancer (csPCa) was defined as a pathological diagnosis of grade group 2 or higher. The proportion of unnecessary biopsies avoided was calculated as the number of non-cancer cases with predictor values below the diagnostic cut-off for csPCa divided by the total number of individuals without cancer. The proportion of grade group 1 prostate cancer diagnoses avoided was defined as the number of patients with predictor values below the cut-off and a diagnosis of grade group 1 prostate cancer divided by the total number of grade group 1 prostate cancer cases.

### 2.5. Statistical Analysis

Continuous variables were presented as medians and interquartile ranges (IQRs) and compared using the Mann–Whitney U test. Categorical variables were presented as proportions and compared using the chi-square test. The diagnostic performance of initial PSA, PSA change, absolute PSA change, PSA density as well as the combination of PSA change and PSA density for detecting prostate cancer or clinically significant prostate cancer was evaluated using receiver operating characteristic (ROC) curves and area under the curve (AUC). AUCs of different predictors were compared using DeLong’s test. Decision curve analysis (DCA) was also performed to assess the clinical utility of each predictor. All statistical analyses were conducted using R software version 4.3.3 (R Foundation for Statistical Computing, Vienna, Austria). A two-tailed *p*-value <0.05 was considered statistically significant.

## 3. Results

### 3.1. Patient Characteristics

A total of 291 patients were included in this study. Among them, 179 (61.5%) had a benign pathology, 35 (12%) had clinically insignificant prostate cancer, and 77 (26.5%) were diagnosed with csPCa. The majority of patients underwent a random biopsy (74.9%). Eleven patients had a history of a previous prostate biopsy (3.8%). Compared to patients without cancer, those diagnosed with cancer were older and had higher PSA levels, less PSA decline, smaller absolute PSA changes, and a higher PSA density. (Table 1). Among patients diagnosed with prostate cancer, those with csPCa had higher PSA, less PSA decline, and a higher PSA density (Table 1). A total of 73 patients underwent an MRI examination. The comparison of PIRADS scores showed significant differences between patients with and without cancer, as well as between those with clinically significant and non-significant cancer.

### 3.2. Comparison of Single Predictive Variable for Diagnosing Prostate Cancer

**Figure 1:**
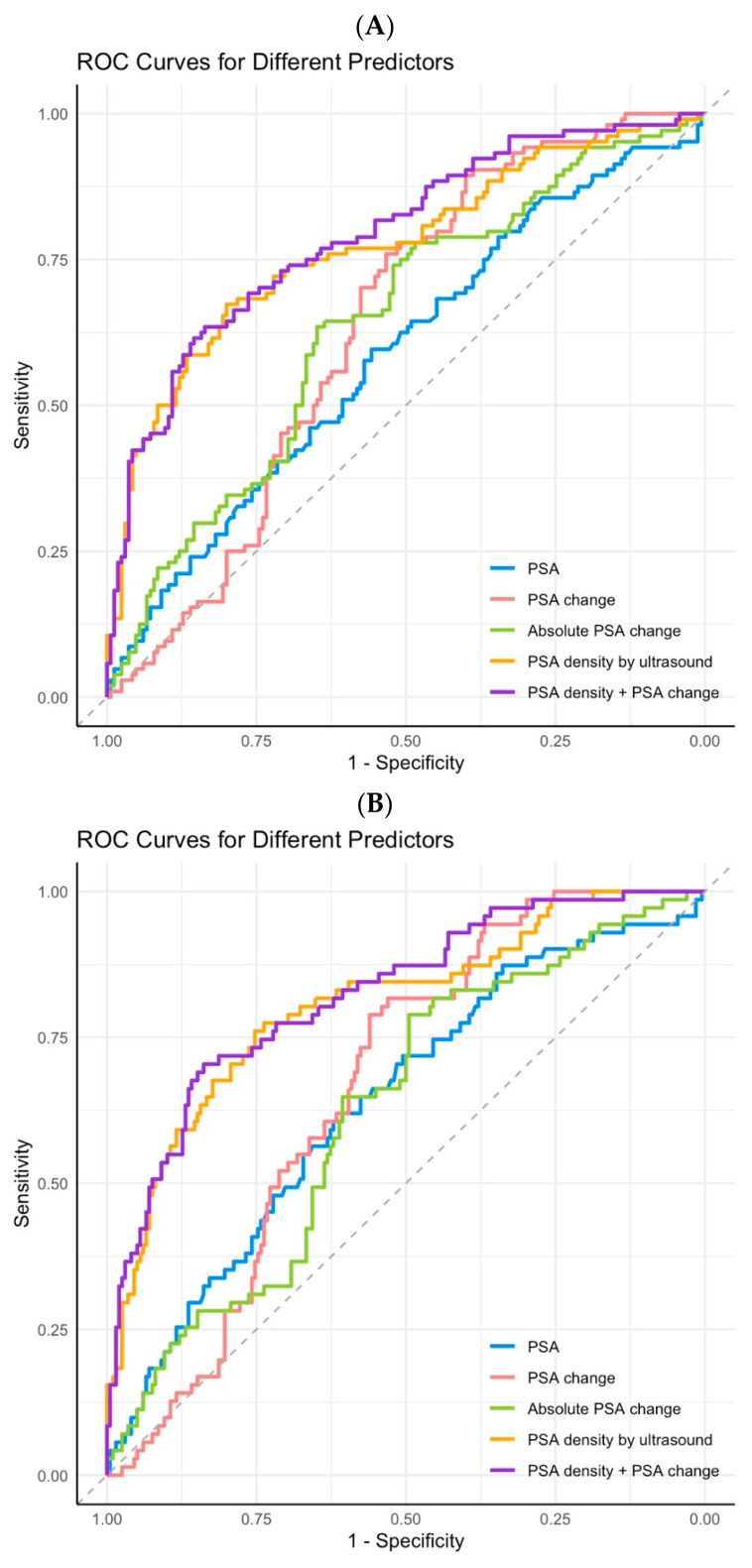
ROC curves of different predictive variables. (**A**) Detection of any-grade cancer. (**B**) Detection of clinically significant cancer.

**Figure 2:**
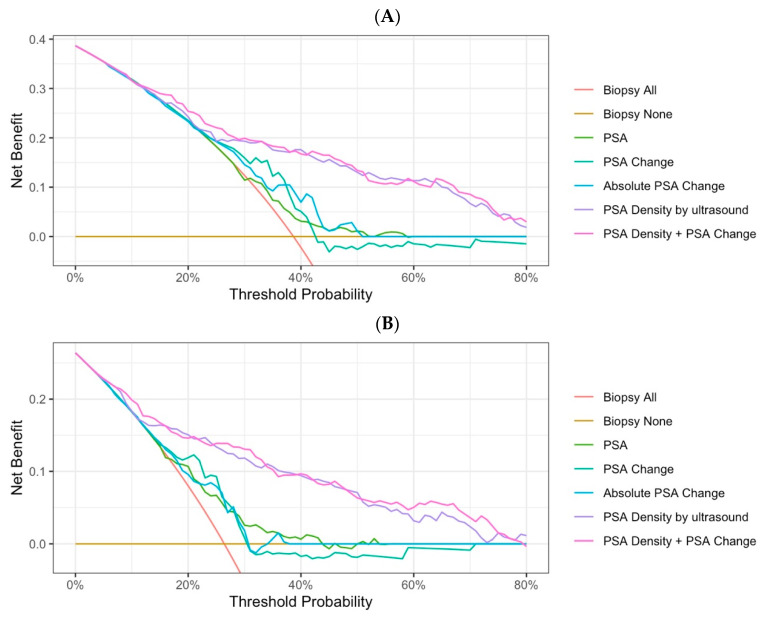
Decision curve analysis of different predictive variables. (**A**) Detection of any-grade cancer. (**B**) Detection of clinically significant cancer.

For predicting any-grade cancer, the PSA density demonstrated the highest diagnostic performance with an AUC of 0.77 (95% CI: 0.71–0.83), which was significantly greater than that of the PSA, PSA change, and absolute PSA change (Table 2, Figure 1A). The DCA also showed that the PSA density provided the greatest net benefit in identifying any-grade cancer (Figure 2A). The AUCs of all variables for predicting prostate cancer decreased as the prostate volume increased. Furthermore, when the prostate volume exceeded 80 mL, the PSA density was no longer a superior predictive variable (Supplementary Table S1). However, regardless of whether MRI was used as a reference for a biopsy, the PSA density consistently outperformed the PSA change as a predictive variable (Supplementary Table S2).

### 3.3. Comparison of Single Predictive Variable for Clinically Significant Prostate Cancer

For the detection of csPCa, the PSA density showed a superior performance with an AUC of 0.81 (95% CI: 0.75–0.87), which is significantly higher than the AUCs of the PSA, PSA change, and absolute PSA change (Table 2, Figure 1B). The3 DCA similarly demonstrated that the PSA density had the highest net benefit in identifying csPCa compared to other predictors (Figure 2B). The difference in the diagnostic accuracy between the PSA density and PSA change diminished as the prostate volume increased (Supplementary Table S1). However, the predictive performance of the PSA density for csPCa was not affected by whether MRI was used as a reference for the biopsy (Supplementary Table S2).

### 3.4. Comparison of PSA Density Derived from TRUS and MRI

**Figure 3:**
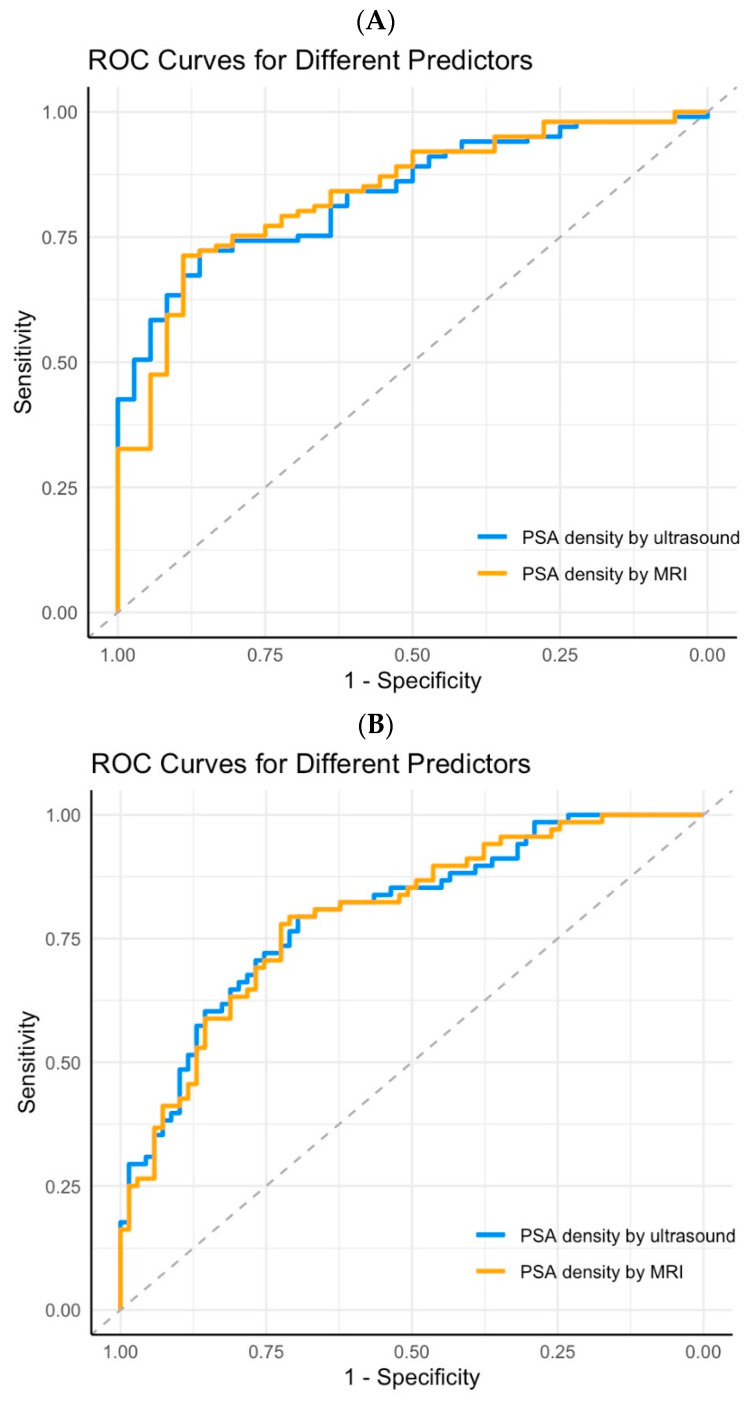
ROC curves of PSA density based on TRUS or MRI. (**A**) Detection of any-grade cancer. (**B**) Detection of clinically significant cancer.

A total of 137 patients had both TRUS- and MRI-based prostate volume measurements. The median prostate volume was 44.3 mL (IQR: 33.8–61.5) when measured by the TRUS and 43.7 mL (IQR: 32.7–62.8) when measured by the MRI. No significant difference was found between the two methods (*p* = 0.656). The diagnostic accuracy of the PSA density calculated using either the TRUS or MRI showed no significant difference in predicting any-grade prostate cancer (*p* = 0.985) or csPCa (*p* = 0.856) (Figure 3).

### 3.5. Impact of Combining PSA Density and PSA Change on Prostate Cancer Diagnosis

For the diagnosis of any-grade cancer, the combination of the PSA density and the PSA change yielded an AUC of 0.79 (95% CI: 0.73–0.84), representing a slight increase compared to the PSA density alone (*p* = 0.068) (Table 2). For csPCa, the combined AUC was 0.82 (95% CI: 0.76–0.88), which is also only marginally higher than the PSA density alone (*p* = 0.049) (Table 2). Nevertheless, the combination yielded increases in the AUC across different prostate volumes and biopsy modalities (Supplementary Tables S1 and S2). In Figure 1 and Figure 2, combining both variables did not result in a significant difference in ROC or DCA analyses compared to the PSA density alone. However, when the PSA density threshold was set at 0.15, omitting biopsies for patients with a PSA decrease greater than 20% helped reduce unnecessary biopsies by 16.4% and the diagnosis of clinically non-significant cancer by 9.1%, without increasing the rate of missed csPCa (1-sensitivity). Similar results were observed when the threshold was set at 0.2 (Table 3). In contrast, when the PSA change threshold was set at a decrease greater than 10% for omitting biopsies, the risk of missed csPCa increased.

## 4. Discussion

This is the first study to compare the diagnostic accuracy of the PSA density and the PSA change for prostate cancer detection. While the PSA density demonstrated a better diagnostic performance than the PSA change, its advantage diminished in patients with larger prostate volumes. However, we found that incorporating the PSA change improved the diagnostic accuracy of the PSA density across all prostate volume subgroups. Furthermore, when the PSA change was considered alongside the PSA density at established biopsy thresholds, unnecessary biopsies could be further reduced without increasing the risk of missing csPCa. These findings suggest that integrating multiple parameters into the biopsy decision-making process can help more accurately identify patients who truly require a prostate biopsy.

Previous studies have demonstrated an association between changes in PSA levels and the prostate cancer diagnosis. Studies by Rosario et al. and De Nunzio et al. both found that a second PSA measurement showing a reduction greater than 20% was associated with a lower likelihood of detecting prostate cancer or csPCa on biopsy [8,10]. Consistent with these findings, our study also observed that patients with prostate cancer or a clinically significant disease tended to exhibit smaller PSA decreases between the two tests. However, the cause of the transient PSA elevation remains unclear in many patients and may be attributable to the intra-individual variation or prostatic inflammation [13]. It is also important to note that some studies have reported patients with confirmed cancer exhibiting PSA levels that return to normal on repeat testing [14,15]. In addition, an antibiotic treatment aimed at reducing potential inflammation does not appear to improve cancer detection [16]. These findings suggest that relying solely on the PSA change for a prostate cancer diagnosis is limited. Nevertheless, the PSA change still has clinical utility. We found that if the PSA declines by more than 20%, patients who meet the biopsy threshold for PSA density are unlikely to have cancer and may safely avoid an unnecessary biopsy.

Our findings, consistent with previous studies, indicate that patients with prostate cancer tend to have significantly smaller prostate volumes compared to those without cancer [8]. Therefore, both the PSA levels and prostate volume should be considered when determining the need for a prostate biopsy. The PSA density adjusts the PSA with respect to the gland size. As a result, patients with elevated PSA but large prostate volumes are less likely to undergo unnecessary biopsies. A prior study based solely on random biopsies reported that the PSA density has a better diagnostic accuracy than PSA alone in detecting prostate cancer and csPCa [17]. In our study, which included patients who underwent a targeted fusion biopsy, we similarly found that the PSA density outperformed PSA. Moreover, our results also demonstrated that the PSA density outperformed both the PSA change and absolute PSA change in diagnostic accuracy, reinforcing the importance of incorporating the prostate volume into risk stratification when deciding whether to perform a biopsy.

An important consideration when using the PSA density is whether the prostate volume should be derived from the TRUS or MRI. Choe et al. reported that the TRUS tends to underestimate the prostate volume compared to MRI, particularly in patients with larger prostates [18]. They also found that the PSA density calculated using MRI-derived volumes demonstrated a better accuracy in detecting the presence of cancer. However, for identifying csPCa, the PSA density derived from the TRUS and MRI showed a comparable performance. The findings of Ye et al. are more consistent with those of our study, indicating no significant difference in prostate volume measurements between the TRUS and MRI [19]. Moreover, the diagnostic accuracy of the PSA density for both any-grade cancer and csPCa was similar regardless of whether the TRUS or MRI was used for the volume estimation. This suggests that the PSA density can be effectively calculated using the TRUS, a simpler and more readily accessible modality in the outpatient setting, to assist with biopsy decisions. Nevertheless, the study by Choe et al. included more patients with larger prostate volumes. Whether the TRUS-derived PSA density remains accurate in such patients warrants further investigation.

Although the PSA density is useful in guiding biopsy decisions, it is not a perfect tool. Our analysis demonstrated that the AUC of the PSA density decreases in patients with larger prostate volumes. A previous study examining the effect of the prostate volume on the accuracy of the PSA density showed similar findings, indicating that both the sensitivity and specificity of the PSA density decline as the prostate volume increases [20]. These results suggest that the PSA density should be combined with other variables to better guide biopsy decisions. Most current studies use the PSA density in conjunction with MRI-based PI-RADS scores for risk stratification, and this combination has consistently been shown to improve diagnostic accuracy [21,22]. In our study, we also found that incorporating the PSA change with the PSA density further increased the diagnostic performance. Compared to MRI, the PSA change is more accessible and less expensive to obtain. The prostate health index (PHI) density has recently been reported to outperform the PSA density in terms of diagnostic accuracy [22]. However, the availability of the PHI varies across regions, making the PSA density a valuable tool for initial prostate cancer risk assessments, particularly in settings where the PHI is not readily accessible.

This study has several limitations. First, due to its retrospective design, the standardization of PSA follow-up intervals and biopsy approaches was not feasible. The diagnostic value of the PSA change may differ depending on the interval between measurements, and further studies are needed to explore this. Second, we did not document whether patients who did not undergo a fusion biopsy had suspicious lesions on ultrasound or whether targeted biopsies were performed for those lesions. If all biopsies had included targeted sampling for any suspicious lesion detected by the MRI or ultrasound, the evaluation of diagnostic variables would have been more precise. Third, not all patients in our cohort underwent both the TRUS and MRI. Therefore, we could not assess whether the MRI-derived information could improve the risk stratification based on the PSA density. Furthermore, since only a subset of patients had both imaging modalities, we were unable to evaluate whether differences in the diagnostic accuracy between the TRUS-based and MRI-based PSA density vary according to the prostate volume. Fourth, this was a single-center study, and findings may not be generalizable to other populations or healthcare settings. Despite these limitations, our findings suggest that combining the PSA change with the PSA density is important for minimizing unnecessary biopsies. Future studies should validate our results in prospective multicenter cohorts, preferably using an MRI-guided biopsy as the reference standard. Additional research is also needed to compare the diagnostic accuracy of different combinations of parameters with the PSA density in order to identify the most effective and cost-efficient approach.

## 5. Conclusions

In men with an initial PSA level between 3 and 20 ng/mL, the PSA density demonstrated a superior diagnostic accuracy compared to the PSA, PSA change, or absolute PSA change for predicting prostate cancer and csPCa. Furthermore, the PSA density derived from either the TRUS or MRI provided a comparable predictive performance for both outcomes. However, the predictive value of the PSA density decreases in patients with larger prostate volumes. Incorporating the PSA change as an additional consideration for a biopsy can help avoid more unnecessary procedures. In patients who meet the biopsy threshold based on the PSA density, a decline of more than 20% in the repeat PSA may be considered a criterion to forgo the biopsy.

## Figures and Tables

**Table 1:** Patient characteristics.

Characteristics	All (N = 291)	Cancer (N = 112)	Non-Cancer (N = 179)	*p*-Value ^a^	Clinically Significant Cancer (N = 77)	Clinically Insignificant Cancer (N = 35)	*p*-Value ^b^
Age, median (IQR)	69 (62–73)	70 (65–76)	68 (62–72)	0.003	70 (64–76)	70 (66–75)	0.732
1st PSA, median (IQR)	8.33 (6.52–11.3)	9.07 (7.05–12)	8.09 (6.3–10.8)	0.025	10.1 (7.59–13)	7.78 (6.32–9.5)	0.004
2nd PSA, median (IQR)	8.31 (6.1–11.2)	9.54 (6.85–12.4)	7.84 (5.8–10.1)	<0.001	10.6 (8.1–13.7)	7.78 (5.75–10.6)	0.001
Interval time (days), median (IQR)	24 (15–39)	23 (15–34)	24 (15–41)	0.195	22 (15–30)	26 (16–39)	0.521
PSA change (%), median (IQR)	−1.1 (−14.3–12.9)	1.9 (−4.2–12.7)	−4.7 (−19.5–12.9)	0.001	5.9 (−2.6–14.8)	−3.2 (−11.4–6.9)	0.013
Absolute PSA change (%), median (IQR)	13.7 (5.9–22.5)	10.4 (3.5–17.1)	16.8 (7.5–24.9)	<0.001	10.2 (3.2–15.9)	10.6 (3.9–22.5)	0.739
TRUS volume, median (IQR) ^c^	48.2 (37–72.2)	39 (31.1–52.3)	58.1 (44.2–77)	<0.001	38.8 (30.3–51)	40.9 (35.4–77.2)	0.156
PSA density by ultrasound, median (IQR) ^c^	0.17 (0.12–0.23)	0.23 (0.17–0.31)	0.15 (0.11–0.19)	<0.001	0.26 (0.2–0.34)	0.18 (0.14–0.23)	0.001
Biopsy method, No. (%)							
Random	218 (74.9%)	89 (79.5%)	129 (72.1%)	0.148	60 (77.9%)	29 (82.9%)	0.603
Cognitive fusion	42 (14.4%)	16 (14.3%)	26 (14.5%)		11 (14.3%)	5 (14.3%)	
Software fusion	31 (10.7%)	7 (6.2%)	24 (13.4)		6 (7.8%)	1 (2.8)	
Total biopsy cores, median (IQR)	12 (12–13)	12 (12–12)	12 (12–13)	0.039	12 (12–12)	12 (12–12)	0.76
Systemic biopsy cores, median (IQR)	12 (12–12)	12 (12–12)	12 (12–12)	0.46	12 (12–12)	12 (12–12)	0.59
Target biopsy cores per target, median (IQR) ^d^	3.4 (3–4)	3 (3–4)	3.5 (3–4)	0.83	3 (3–4)	5 (3.6–5)	0.53
PIRADS, No. (%) ^e^							
2	16 (21.9%)	1 (4.3%)	15 (30%)	<0.001	0 (0%)	1 (16.7%)	0.019
3	37 (50.7%)	4 (17.4%)	33 (66%)		1 (5.9%)	3 (50%)	
4	12 (16.4%)	10 (43.5%)	2 (4%)		9 (52.9%)	1 (16.7%)	
5	8 (11%)	8 (34.8%)	0 (0%)		7 (41.2%)	1 (16.7%)	

^a^ *p*-value between cancer and non-cancer patients. ^b^ *p*-value between clinically significant and clinically insignificant cancer patients. ^c^ Only 269 patients had TRUS volume, and 104 of which were diagnosed with cancer. ^d^ Only 54 patients had documented target biopsy cores, and 13 of which were diagnosed with cancer. ^e^ Only 73 patients had underwent MRI, and 23 of which were diagnosed with cancer.

**Table 2:** Diagnostic performance of different predictive variables.

Variables	Detection of Any-Grade Cancer			Detection of Clinically Significant Cancer		
	AUC	95% CI	*p*-Value	AUC	95% CI	*p*-Value
PSA	0.59	0.52–0.66	<0.001	0.64	0.56–0.71	<0.001
PSA change	0.64	0.57–0.70	0.003	0.67	0.60–0.73	0.002
Absolute PSA change	0.64	0.57–0.71	0.004	0.63	0.55–0.70	<0.001
PSA density + PSA change	0.79	0.73–0.84	0.068	0.82	0.76–0.88	0.049
PSA density	0.77	0.71–0.83	Reference	0.81	0.75–0.87	Reference

**Table 3:** Effect of combining PSA density and PSA change on diagnostic accuracy.

Cut-Off for Biopsy Decision	Sensitivity	Specificity	Unnecessary Biopsy Avoided	Grade Group 1 Cancer Avoided
PSA density ≥0.15	84.5%	49.5%	50.3%	39.4%
PSA density ≥0.15 + PSA does not decrease >20%	84.5%	63.6%	66.7%	48.5%
PSA density ≥0.15 + PSA does not decrease >10%	73.2%	72.2%	75.8%	54.5%
PSA density ≥0.2	73.2%	75.8%	80.6%	51.5%
PSA density ≥0.2 + PSA does not decrease >20%	73.2%	81.8%	86.1%	60.6%
PSA density ≥0.2 + PSA does not decrease >10%	64.8%	86.9%	90.9%	66.7%

## Data Availability

The data sets generated during and/or analyzed during the current study are available from the corresponding author on reasonable request.

## Supplementary Materials

The following supporting information can be downloaded at https://www.mdpi.com/article/10.3390/diagnostics15162027/s1. Table S1: Diagnostic performance of different predictive variables stratified by prostate volume, Table S2: Diagnostic performance of different predictive variables stratified by biopsy approach.
