# Supplementary material for: Integrating PSA Change with PSA Density Enhances Diagnostic Accuracy and Helps Avoid Unnecessary Prostate Biopsies
Source: Diagnostics (Basel). 2025 Aug 13;15(16):2027. doi: 10.3390/diagnostics15162027 (PMC12385582; doi:10.3390/diagnostics15162027)
Supplement: Supplementary file 1 [file diagnostics-15-02027-s001.zip › diagnostics-3772614-supplementary.pdf]

Supplement Table S1 Diagnostic performance of different predictive variables stratified by prostate volume

Variables	Detection of any grade cancer			Detection of clinically significant caner		
	AUC	95% CI	p-value	AUC	95% CI	p-value
Prostate volume <30ml (N=29)						
PSA	0.66	0.41–0.91	0.008	0.7	0.51–0.9	0.015
PSA change	0.64	0.37–0.91	0.51	0.63	0.41–0.84	0.16
Absolute PSA change	0.69	0.44–0.93	0.54	0.59	0.37–0.81	0.065
PSA density + PSA change	0.77	0.55-0.99	0.77	0.82	0.66–0.97	0.83
PSA density	0.76	0.53–0.98	Reference	0.81	0.66–0.97	Reference
Prostate volume 30 – 80ml (N=194)						
PSA	0.64	0.56–0.73	<0.001	0.7	0.61–0.79	0.019
PSA change	0.65	0.58–0.73	0.06	0.7	0.62–0.77	0.18
Absolute PSA change	0.63	0.55–0.71	0.03	0.6	0.51–0.69	0.011
PSA density + PSA change	0.77	0.71–0.84	0.1	0.79	0.72–0.87	0.083
PSA density	0.75	0.68–0.82	Reference	0.77	0.69–0.85	Reference
Prostate volume >80ml (N=46)						
PSA	0.53	0.31–0.75	0.95	0.37	0.01–0.73	0.29
PSA change	0.62	0.43–0.82	0.62	0.65	0.48–0.82	0.79
Absolute PSA change	0.58	0.35–0.8	0.73	0.72	0.48–0.96	0.85
PSA density + PSA change	0.68	0.47–0.88	0.3	0.74	0.51–0.97	0.3
PSA density	0.54	0.33–0.76	Reference	0.71	0.44–0.97	Reference

Supplement Table S2 Diagnostic performance of different predictive variables stratified by biopsy approach

Variables	Detection of any grade cancer			Detection of clinically significant caner		
	AUC	95% CI	p-value	AUC	95% CI	p-value
Random biopsy (N=215)						
PSA	0.57	0.49–0.65	<0.001	0.64	0.56–0.72	<0.001
PSA change	0.62	0.54–0.69	0.02	0.66	0.58–0.73	0.015
Absolute PSA change	0.65	0.57–0.72	0.07	0.63	0.55–0.71	0.005
PSA density + PSA change	0.76	0.7–0.83	0.08	0.81	0.74–0.87	0.07
PSA density	0.74	0.67–0.81	Reference	0.79	0.71–0.86	Reference
Image guided biopsy (N=54)						
PSA	0.65	0.5–0.8	0.009	0.65	0.48–0.83	0.006
PSA change	0.72	0.59–0.86	0.12	0.72	0.59–0.86	0.048
Absolute PSA change	0.62	0.44–0.8	0.017	0.6	0.39–0.81	0.041
PSA density + PSA change	0.9	0.82–1	0.42	0.89	0.78–1	0.76
PSA density	0.87	0.75–1	Reference	0.88	0.77–1	Reference
